# Meta-analysis of the efficacy and safety of SGLT-2 inhibitors in patients with heart failure and type 2 diabetes mellitus

**DOI:** 10.1097/MD.0000000000042196

**Published:** 2025-05-02

**Authors:** Xinliang Yao, Han Zhang, Xueli Lu

**Affiliations:** a Department of Cardiology, Huaihe Hospital of Henan University, Gulou District, Kaifeng, Henan Province, China.

**Keywords:** efficacy and safety, heart failure, meta-analysis, SGLT-2 inhibitors, type 2 diabetes mellitus

## Abstract

**Background:**

To investigate the efficacy and safety of sodium-glucose cotransporter 2 (SGLT-2) inhibitors in patients with heart failure (HF) and type 2 diabetes mellitus (T2DM).

**Methods:**

A manual search was conducted in 3 prestigious English databases, Cochrane Library, PubMed, and Web of Science, for studies published within the last decade, from July 2014 to July 2024. The extracted literature was synthesized to analyze the efficacy outcomes, survival prognostic indicators, and safety profiles of SGLT-2 inhibitors in patients with HF and T2DM. The Cochrane bias risk assessment scale was used as a tool to evaluate the quality of the literature, and Review Manager 5.4 software was used to create the bias risk chart. Data analysis and merging were completed with the help of Review Manager 5.4 and Stata 15.0 statistical software.

**Results:**

Twelve studies encompassing 9509 patients were included in the meta-analysis. The results revealed that compared to the control group, the SGLT-2 inhibitor-treated group demonstrated significantly greater reductions in left ventricular end-diastolic volume index [mean difference (MD) = -7.25, 95% confidence intervals [95% CI] (-9.83, -4.67)], brain natriuretic peptide levels [MD = -36.96, 95% CI (-63.51, -10.41)], and N-terminal pro-brain natriuretic peptide [MD = -519.27, 95% CI (-660.77, -377.78)]. Furthermore, the SGLT-2 inhibitor-treated group exhibited significantly higher increases in Kansas City Cardiomyopathy Questionnaire scores [MD = 3.32, 95% CI (3.30, 3.34)], indicating improved quality of life. Additionally, the incidence of adverse events was significantly lower in the SGLT-2 inhibitor-treated group compared to the control group [OR = 0.78, 95% CI (0.69, 0.88)]. The pooled results of the meta-analysis indicated that SGLT-2 inhibitor therapy reduced the risk of cardiovascular death or HF hospitalization by 23%, the risk of cardiovascular death by 19%, and the risk of all-cause mortality by 9%.

**Conclusion:**

SGLT-2 inhibitor therapy significantly reduced the risks of all-cause mortality, cardiovascular death, and hospitalization for HF in patients with HF and T2DM. Additionally, SGLT-2 inhibitors significantly improve cardiac function, decrease the incidence of adverse events, and enhance the quality of life in these patients.

## 1. Introduction

The dual burden of heart failure (HF) and type 2 diabetes mellitus (T2DM) has emerged as a growing health concern globally.^[1,2]^ HF is one of the most prevalent cardiovascular complications in T2DM patients, with a notably elevated incidence and poorer prognosis.^[3,4]^ Approximately 40% of HF patients concurrently suffer from T2DM, a proportion that continues to escalate, posing significant challenges to patients’ quality of life and survival prognosis.^[5]^ Consequently, exploring effective therapeutic strategies to improve clinical outcomes in this patient population is of paramount importance.

Sodium-glucose cotransporter 2 (SGLT-2) inhibitors have emerged as key agents in the management of T2DM, primarily by inhibiting the SGLT-2 in the kidneys, leading to enhanced glucose excretion and improved glycemic control. Recent studies have identified additional mechanisms by which these drugs may exert beneficial effects on cardiovascular and renal health. Studies^[6–8]^ have suggested that SGLT-2 inhibitors may reduce oxidative stress, improve endothelial function, and offer protection against HF and chronic kidney disease in diabetic and non-diabetic populations.

In recent years, SGLT-2 inhibitors, a novel class of oral hypoglycemic agents, have shown unique advantages in the treatment of diabetes and its complications.^[9,10]^ By inhibiting the reabsorption of glucose in the proximal tubules of the kidney, SGLT-2 inhibitors facilitate the excretion of glucose in the urine, thereby achieving glycemic control.^[11]^ This mechanism not only ensures effective blood glucose management, but also significantly reduces the risk of cardiovascular and renal diseases.^[12]^ In particular, the application of SGLT-2 inhibitors in the treatment of patients with HF and T2DM has garnered considerable attention.^[13]^

Several large-scale clinical trials have corroborated the remarkable efficacy of SGLT-2 inhibitors in reducing the risk of HF hospitalization, cardiovascular mortality, and improving renal outcomes.^[14–16]^ However, due to variations in study populations, design protocols, and follow-up durations, heterogeneity exists among these findings. Therefore, a systematic evaluation of the efficacy and safety of SGLT-2 inhibitors in patients with HF and T2DM is essential to guide clinical practice. This study aims to comprehensively assess the existing controlled trials on SGLT-2 inhibitor therapy for HF and T2DM patients through a meta-analysis approach, aiming to clarify their efficacy and safety, and ultimately provide more comprehensive and scientific evidence to support the treatment of HF and T2DM patients.

## 2. Methods

### 2.1. Literature search strategy

Given the international and specialized nature of the research, a manual search was conducted across 3 authoritative English databases: Cochrane Library, PubMed, and Web of Science. To ensure the timeliness and capture the latest advancements, the search timeframe was precisely set to cover the past decade, from July 2014 to July 2024. A combination of subject terms and free-text words was used to enhance the sensitivity and specificity of the search. The primary search keywords included “heart failure OR HF,” “Sodium-Glucose Transporter 2 Inhibitors OR SGLT-2 Inhibitors,” “Canagliflozin OR Dapagliflozin OR Empagliflozin OR Luseogliflozin,” and “type 2 diabetes.” All retrieved articles were managed using EndNote 21 software.

A systematic literature search was performed to identify studies that explored the mechanisms of SGLT-2 inhibitors in T2DM and their associated effects on cardiovascular and renal outcomes. Studies included in this review were selected based on their relevance to the primary effects of SGLT-2 inhibitors and their contributions to understanding secondary mechanisms such as oxidative stress reduction, endothelial function improvement, and HF protection.^[17–19]^

### 2.2. Inclusion and exclusion criteria

Inclusion criteria: (1) study population: studies explicitly including patients with HFcombined with T2DM as the research subjects. (2) Intervention measures: studies utilizing at least 1 SGLT-2 inhibitor (Canagliflozin, Dapagliflozin, Empagliflozin, or Luseogliflozin) as the intervention. (3) Outcome indicators: studies reporting any of the following outcome indicators: ① efficacy indicators: N-terminal pro-brain natriuretic peptide (NT-proBNP), brain natriuretic peptide (BNP), left ventricular end-diastolic volume index (LVEDVI); Kansas City Cardiomyopathy Questionnaire (KCCQ); ② survival prognosis indicators: risk of HF hospitalization (HHF), risk of cardiovascular death, risk of all-cause death, risk of composite outcome of HF hospitalization or cardiovascular death; ③ safety indicators: incidence of adverse events. (4) Language limitation: only English literature was included to ensure the accuracy and accessibility of the research information.

Exclusion criteria: (1) reviews, conference abstracts, commentary articles, etc. (2) Multiple publications of the same study, which were identified and excluded using the deduplication function of EndNote 21 and manual inspection. (3) Non-randomized controlled trials, case reports, and observational studies. (4) Studies that did not explicitly mention HF combined with T2DM or included patients with other disease states. (5) Animal and in vitro experiments as well as pharmacokinetic studies. (6) Studies with incomplete or unavailable data.

### 2.3. Literature screening and data extraction

Two independent researchers conducted the database search and initial screening process separately, rapidly reviewing the titles and abstracts of all retrieved articles. Based on the predefined inclusion and exclusion criteria, they preliminarily excluded those that obviously did not meet the conditions. For studies repeatedly published across multiple databases or in different issues in the same journal, the 1 with the most complete and detailed data was preferentially selected to avoid duplicate calculations and analyses. For articles that potentially met the inclusion criteria after the initial screening, both researchers thoroughly read the full text. During this stage, meticulous screening was conducted, strictly adhering to the established inclusion and exclusion criteria. This process required researchers to thoroughly understand the study design, methodology, results, and conclusions of the literature to ensure that the quality of the included studies met analytical requirements. In cases of disagreement during the screening process, a third researcher was invited to join the discussion to jointly resolve the issue, ensuring the objectivity and consistency of the screening results.

For the successfully included studies, data extraction was performed. The extracted content included, but was not limited to, the following: authors and publication year of the literature for tracing the research background and source; quality evaluation information such as the use of randomization, blinding, and sample size to assess the scientific rigor and credibility of the study; baseline characteristics of the study population, including age and follow-up duration, for inter-group comparisons; specific intervention protocols and measures for the experimental and control groups to clarify the intervention effects of the study; and relevant cardiac function and prognosis outcome indicators after treatment, such as cardiac function improvement, incidence of cardiovascular events, and incidence of adverse events, as key data for analyzing efficacy and safety.

### 2.4. Quality assessment of literature

The Cochrane Risk of Bias Assessment Tool was employed, utilizing Review Manager 5.4 software to create a risk of bias graph. Studies were evaluated holistically based on the 7 domains of Cochrane Risk of Bias. If all domains were rated as “low risk,” the study was deemed overall as “low risk.” If some domains were rated as “unclear risk” but no “high risk” domains existed, the overall evaluation was “some concerns.” Any study with a “high risk” rating in any domain was deemed “high risk.”

### 2.5. Statistical analysis

For continuous variables and survival data, standardized mean differences and hazard ratios (HR) were adopted as effect size indicators. Data analysis was completed using Review Manager 5.4 and Stata 15.0 statistical software. To present the analysis results more intuitively, the logarithm of the HR (log HR) and its standard error were calculated. Additionally, all effect sizes were expressed with 95% confidence intervals (95% CI) to enhance the reliability of the results. When the *P*-value of the *Q* test was >.05, indicating no significant heterogeneity among the studies, a fixed-effects model was used to pool the effect sizes for cases with no or low heterogeneity. In cases of moderate-to-high heterogeneity among studies, a random-effects model was employed to combine the data results.

## 3. Results

### 3.1. Literature search results

#### 3.1.1. Literature inclusion process

**Figure 1.**
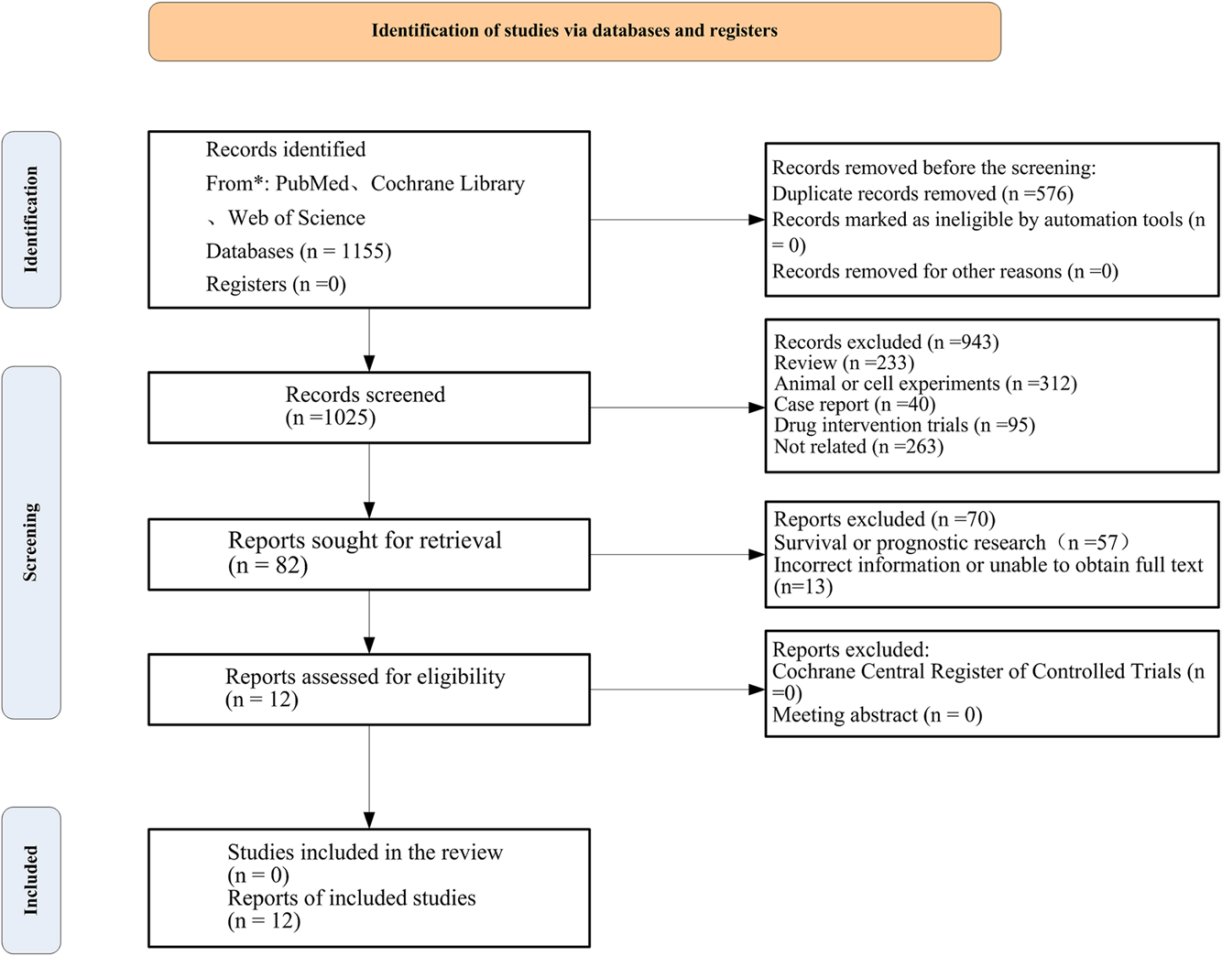
Literature inclusion process.

PubMed, Web of Science database returned 169 articles, and Library of Congress databases yielded 912, 169, and 71 articles, respectively. Three additional articles were manually identified through supplementary searches on Google Scholar, resulting in an initial total of 1155 articles. After removing duplicates, 1025 articles remained. Following the exclusion of 156 irrelevant articles and further screening of titles and abstracts, 774 articles were discarded, leaving 82 articles for full-text review. Upon reviewing the full texts, articles with incomplete data, inconsistent outcomes, or non-compliant study subjects were excluded, ultimately resulting in the inclusion of 12 articles, as illustrated in Figure 1.

#### 3.1.2. General characteristics of included studies

Twelve studies^[20–31]^ were included, encompassing 9509 patients, with 4828 patients in the experimental group (treated with SGLT-2 inhibitors) and 4681 patients in the control group. The average age of the study participants was over 66 years, classifying them as elderly. Among the included studies, empagliflozin was the most commonly used SGLT-2 inhibitor in the experimental group, followed by canagliflozin. The control group received either placebo or standard diabetic therapy. Detailed information is presented in Table 1.

**Table 1:** General characteristics of included studies.

First author, year of publication	Follow-up time	Number of experimental group members	Number of control group members	Experimental group drugs	Control group drugs	Age of experimental group	Age of control group	Outcome measures
Afshani MR (2024)^[14]^	6 months	52	52	Engagliflozin	Standard diabetic therapy	–	–	A1
Anker SD (2021)^[15]^	12 months	927	929	Empagliflozin	Placebo	66.8 ± 10.0	66.6 ± 10.3	A3, B1, B2
Filippatos G (2022)^[16]^	–	1466	1472	Empagliflozin	Placebo	–	–	B1, B2, B3, C
Lee MMY (2021)^[17]^	36 weeks	52	53	Empagliflozin	Placebo	68.2 (11.7)	69.2 (10.6)	A1, A3, A4
Petrie MC (2020)^[18]^	12 months	1075	1064	Dapagliflozin	Placebo	66.3 (9.9)	66.7 (9.8)	A3, B1, B2, B3
Radholm K (2018)^[19]^	188.2 weeks	803	658	Canagliflozin	Placebo	64.1 (8.3)	63.4 (8.3)	B1, B2
Sarraju A (2021)^[20]^	24 weeks	113	120	Canagliflozin	Glimepiride	68.3 ± 9.8	68.9 ± 10.4	B1, B3
Tamaki S (2021)^[21]^	–	30	29	Empagliflozin	Standard diabetic therapy	80 (77–83)	82 (75–84)	A2, A4
Tanaka A (2022)^[22]^	24 weeks	64	65	Canaglifozin	Glimepiride	68.7 ± 9.6	69.4 ± 9.3	A4
Ueda T (2021)^[23]^	–	42	40	Canagliflozin	standard diabetic therapy	76.5 ± 6.4	75.9 ± 5.8	A2, C
Xanthopoulos A (2023)^[24]^	12 months	60	58	Dapagliflozin	Standard diabetic therapy	67.00 (62.25–73.00)	73.50 (66.50–78.25)	A1, A2
Yoshihara F (2023)^[25]^	24 months	144	141	Dapagliflozin	Standard diabetic therapy	72.1 (9.4)	72.2 (9.5)	B2, B3, C

Among the observation indicators, A1 to A4 are left ventricular end-diastolic volume index, BNP, KCCQ, and NT-proBNP, respectively; B1 to B3 are time to first event of cardiovascular death or HHF, time to cardiovascular death, and time to all-cause mortality, respectively; C is the incidence of adverse reactions.

BNP = brain natriuretic peptide, KCCQ = Kansas City Cardiomyopathy Questionnaire, NT-proBNP = N-terminal pro-brain natriuretic peptide.

#### 3.1.3. Quality of literature

**Figure 2.**
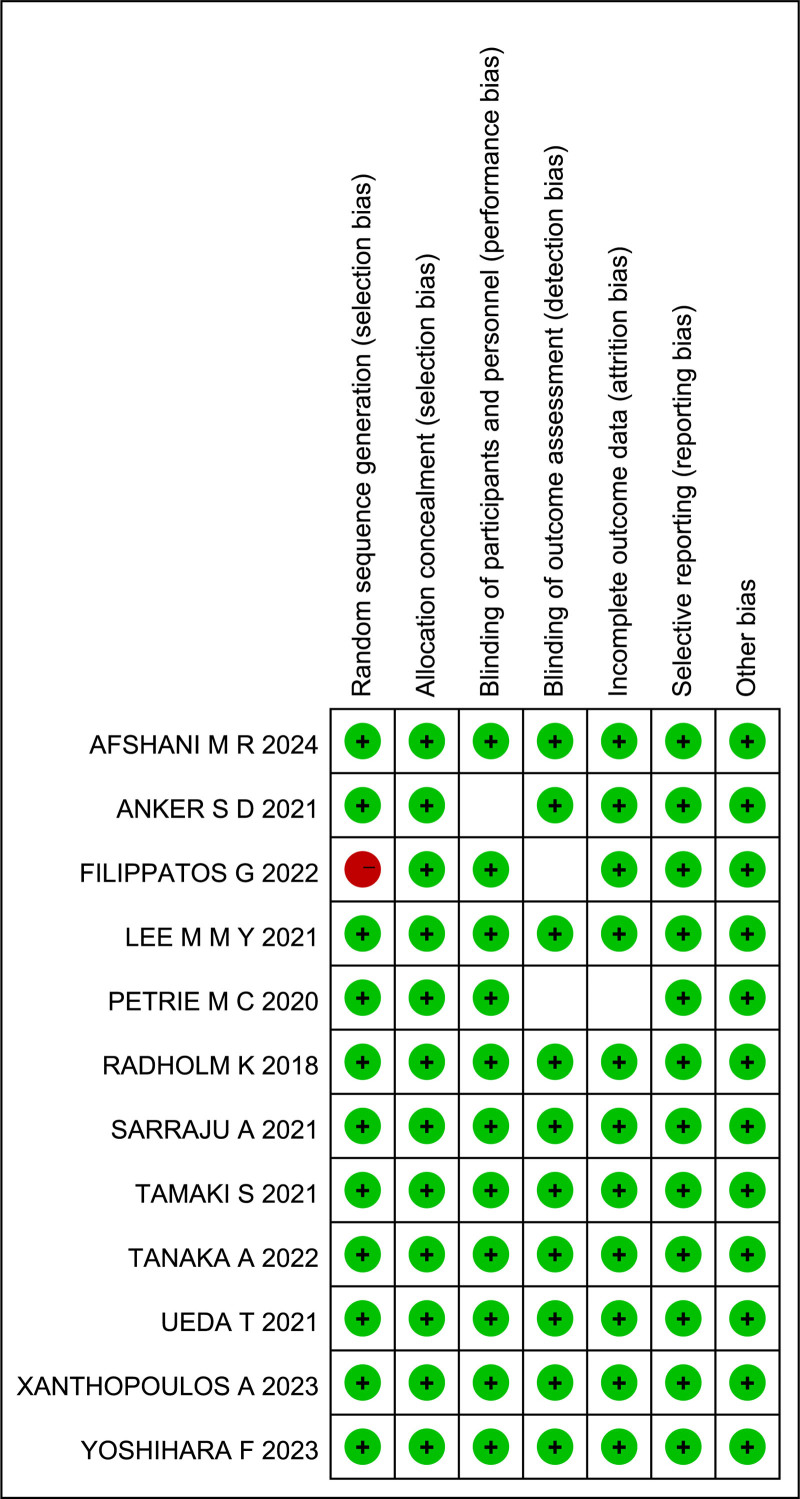
Quality evaluation of included literature.

The study by Filippatos et al^[22]^ was identified as high-risk due to the presence of a high-risk in random sequence generation (selection bias), while Anker SD^[21]^ and Petrie MC^[24]^ were classified as medium-risk due to unknown risks in blinding of outcome assessment (detection bias). The remaining 9 studies^[20,23,25–31]^ were deemed low-risk across all 7 dimensions and were considered high-quality, low-risk literature. Therefore, the majority of the included studies were low-risk, indicating a high overall quality of the included literature, as shown in Figure 2.

### 3.2. META-analysis results

#### 3.2.1. Efficacy outcomes META-analysis results

**Figure 3.**
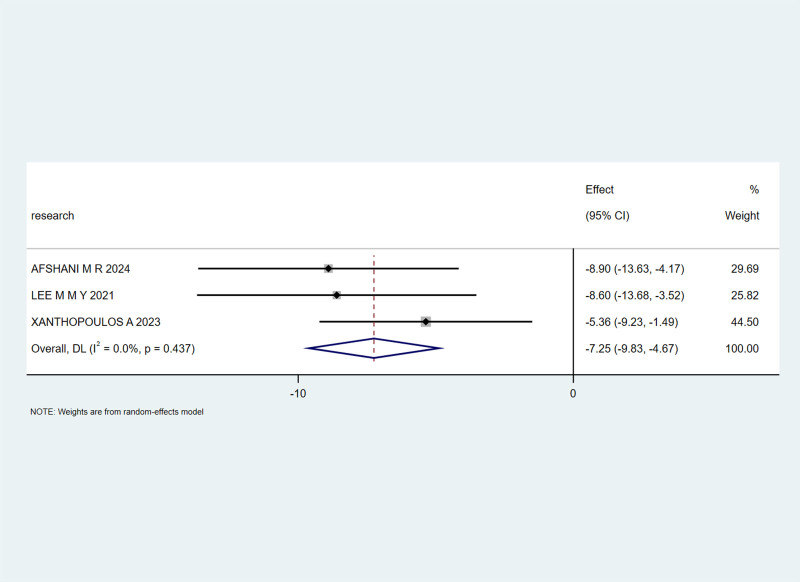
The effect of SGLT-2 inhibitors on the LVEDVI in patients. LVEDVI = left ventricular end-diastolic volume index, SGLT-2 = sodium-glucose cotransporter 2.

Three studies^[20,23,30]^ reported LVEDVI. With an I² heterogeneity of 0%, a fixed-effects model was chosen. The meta-analysis revealed a combined effect size of mean difference (MD) = -7.25, 95% CI (-9.83, -4.67), Z = 5.51, *P* < .00001, indicating a statistically significant reduction in LVEDVI in the experimental group compared to that in the control group, as shown in Figure 3.

**Figure 4.**
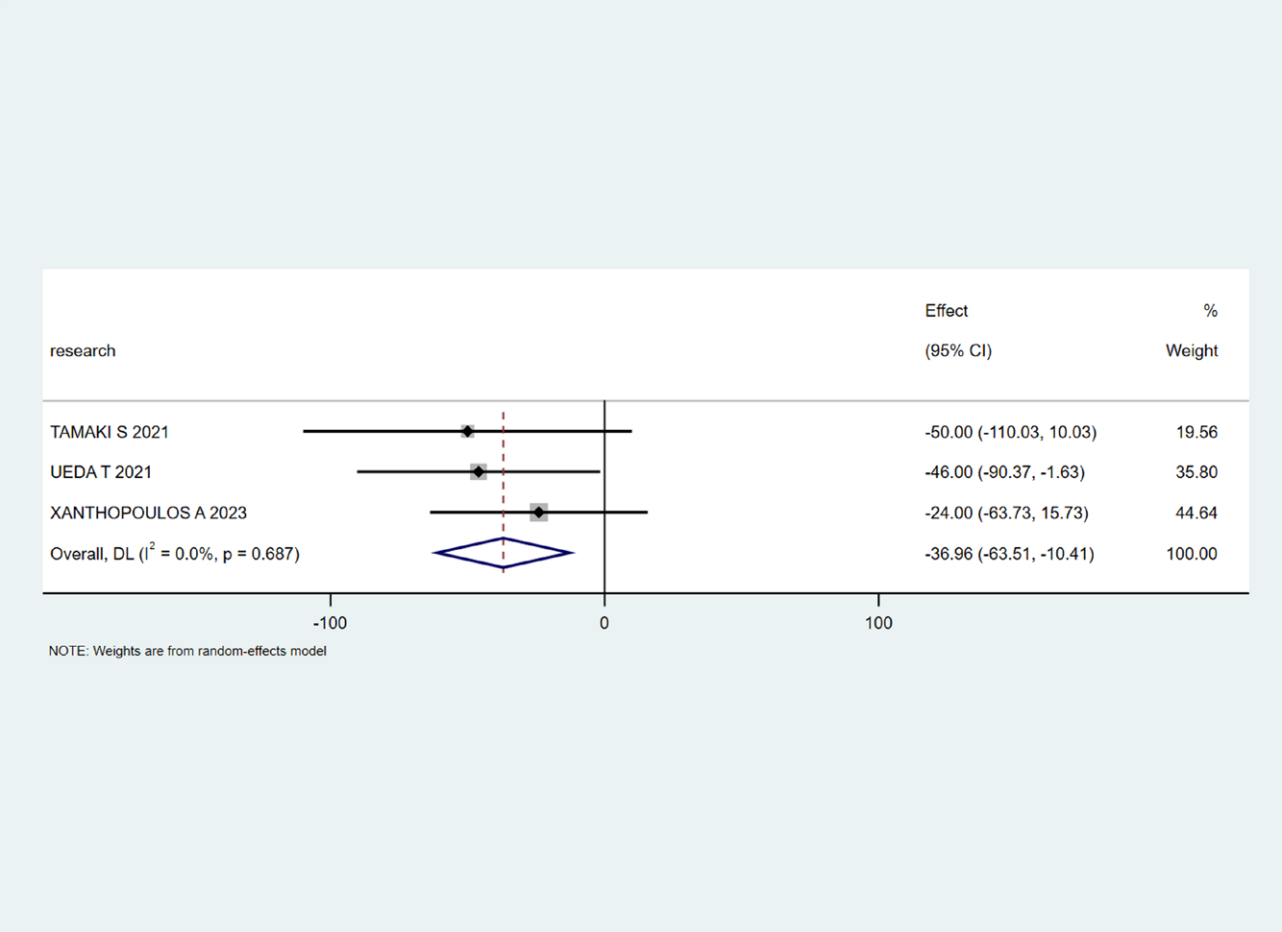
The effect of SGLT-2 inhibitors on BNP levels in patients. BNP = brain natriuretic peptide, SGLT-2 = sodium-glucose cotransporter 2.

Three studies^[27,29,30]^ mentioned the BNP marker. With an I² heterogeneity of 0%, a fixed-effects model was applied. The meta-analysis showed a combined effect size of MD = -36.96, 95% CI (-63.51, -10.41), Z = 2.73, *P* < .05, indicating a significant decrease in BNP levels in the experimental group compared to the control group, with statistical significance, as shown in Figure 4.

**Figure 5.**
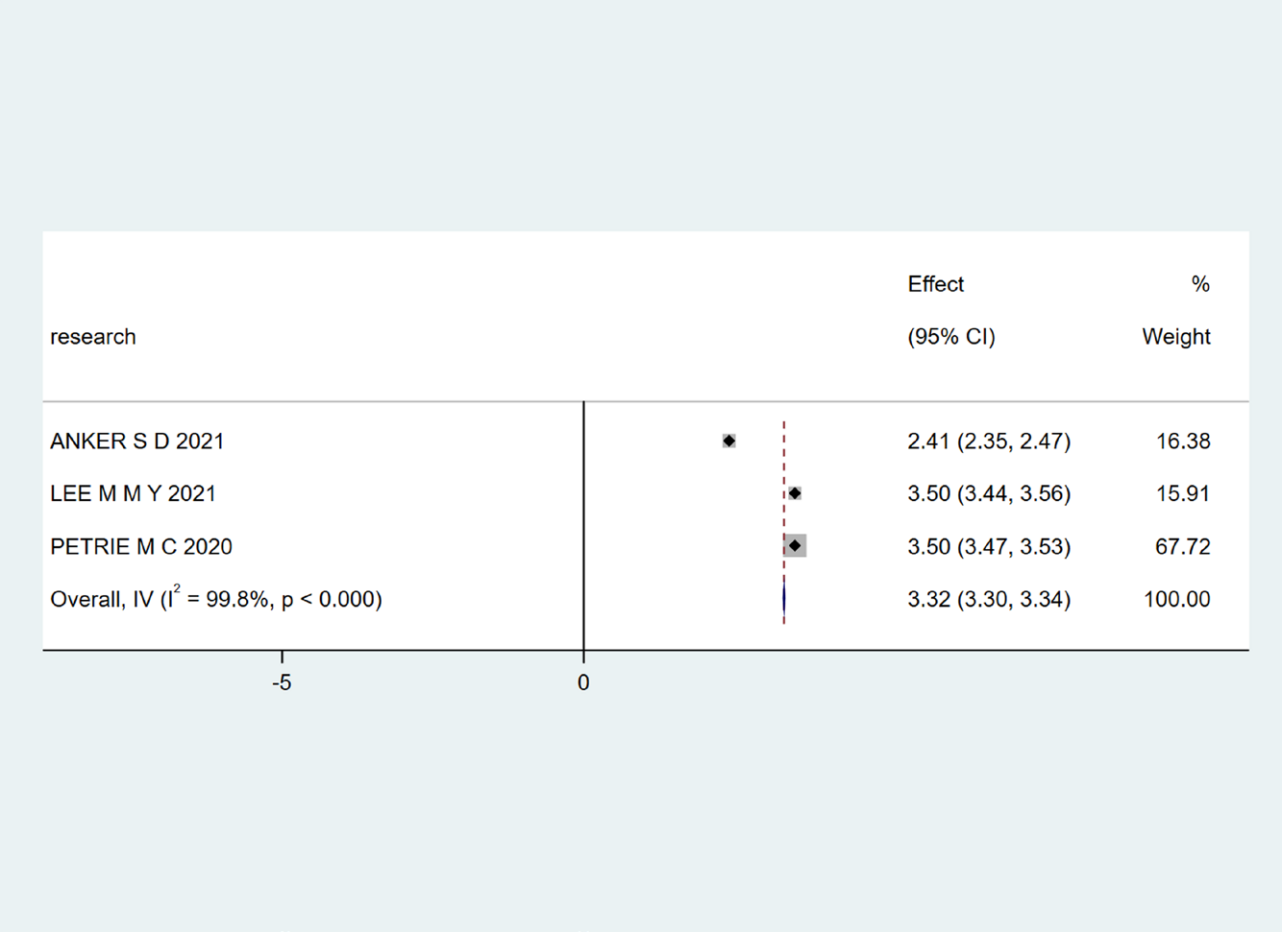
The effect of SGLT-2 inhibitors on patient KCCQ scores. KCCQ = Kansas City Cardiomyopathy Questionnaire, SGLT-2 = sodium-glucose cotransporter 2.

Three studies^[21,23,24]^ reported KCCQ scores. Due to an I² heterogeneity of 100%, a random-effects model was chosen. The meta-analysis yielded a combined effect size of MD = 3.32, 95% CI (3.30, 3.34), Z = 278.41, *P* < .00001, indicating a significant improvement in KCCQ scores in the experimental group compared to the control group, with statistical significance, as shown in Figure 5.

**Figure 6.**
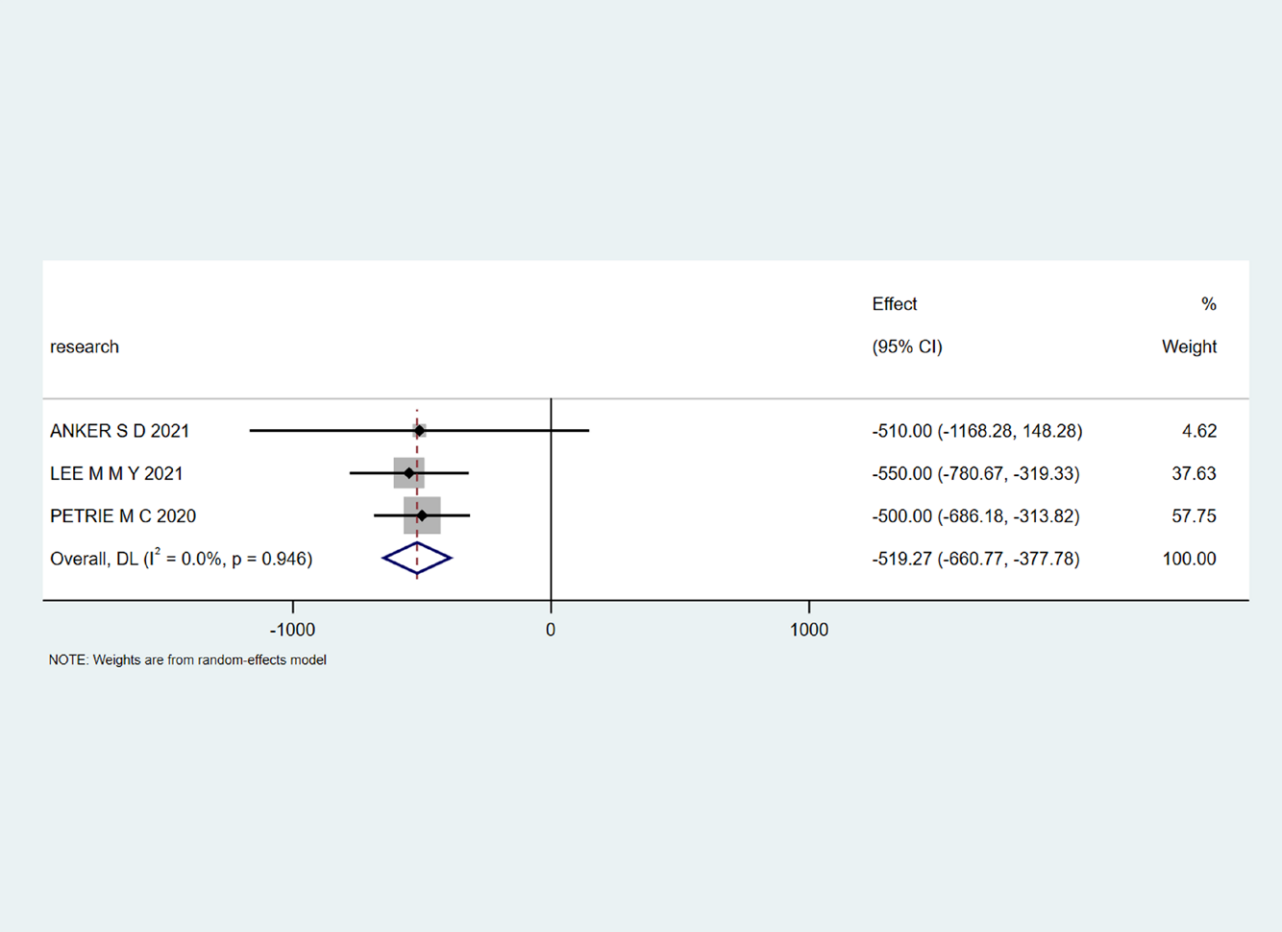
Effect of SGLT-2 inhibitors on NT-proBNP levels in patients. NT-proBNP = N-terminal pro-brain natriuretic peptide, SGLT-2 = sodium-glucose cotransporter 2.

Three studies^[23,27,28]^ reported the NT-proBNP levels. With an I² heterogeneity of 0%, a fixed-effects model was applied. The meta-analysis revealed a combined effect size of MD = -519.27, 95% CI (-660.77, -377.78), Z = 7.19, *P* < .00001, indicating a significant reduction in NT-proBNP levels in the experimental group compared to the control group, with statistical significance, as shown in Figure 6.

#### 3.2.2. Prognostic outcomes META-analysis results

**Figure 7.**
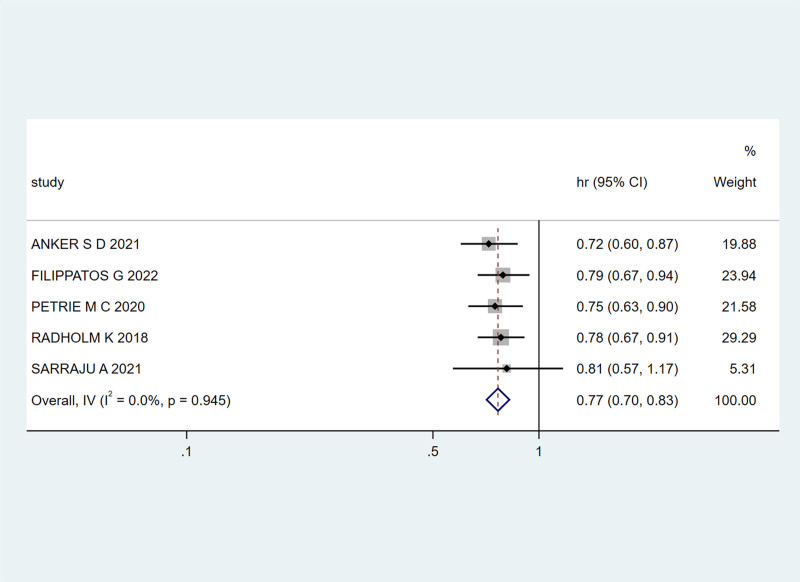
The effect of SGLT-2 inhibitors on the risk of cardiovascular disease death or hospitalization for heart failure in patients. SGLT-2 = sodium-glucose cotransporter 2.

Five studies^[21,22,24–26]^ addressed the time to the first event of cardiovascular death or HHF in patients with HF and T2DM treated with SGLT-2 inhibitors. As there was no heterogeneity among the studies (I² = 0.0%, *P* = .945), a fixed-effects model was used. The combined HR and 95% CI were 0.77 (0.70, 0.83), indicating a 23% reduction in cardiovascular death or HHF events with SGLT-2 inhibitor treatment (Fig. 7).

**Figure 8.**
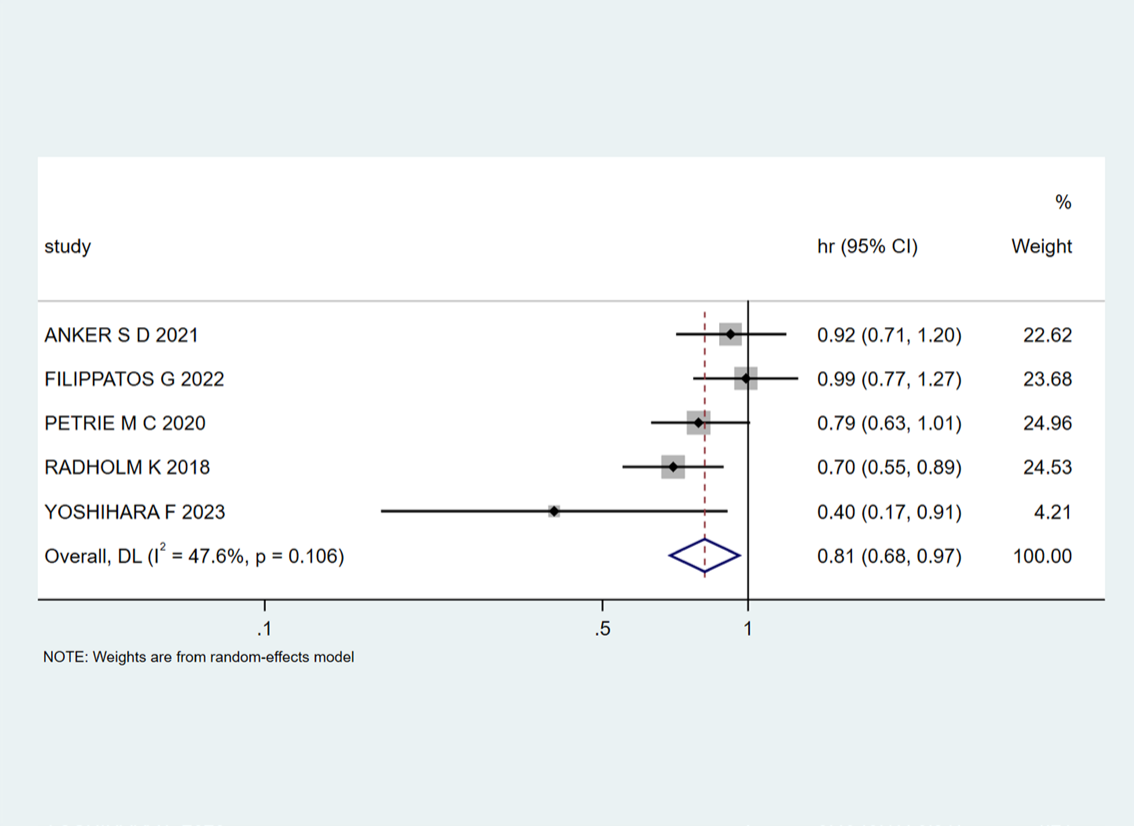
Impact of SGLT-2 inhibitors on the risk of cardiovascular disease mortality in patients. SGLT-2 = sodium-glucose cotransporter 2.

Five studies^[21,22,24,25,31]^ investigated the time to cardiovascular death in patients with HF and T2DM treated with SGLT-2 inhibitors. Moderate heterogeneity was observed (I² = 47.6%, *P* = .106), prompting the use of a random-effects model. The combined HR and 95% CI were 0.81 (0.68, 0.97), indicating a 19% reduction in cardiovascular death events with SGLT-2 inhibitor treatment, as shown in Figure 8.

**Figure 9.**
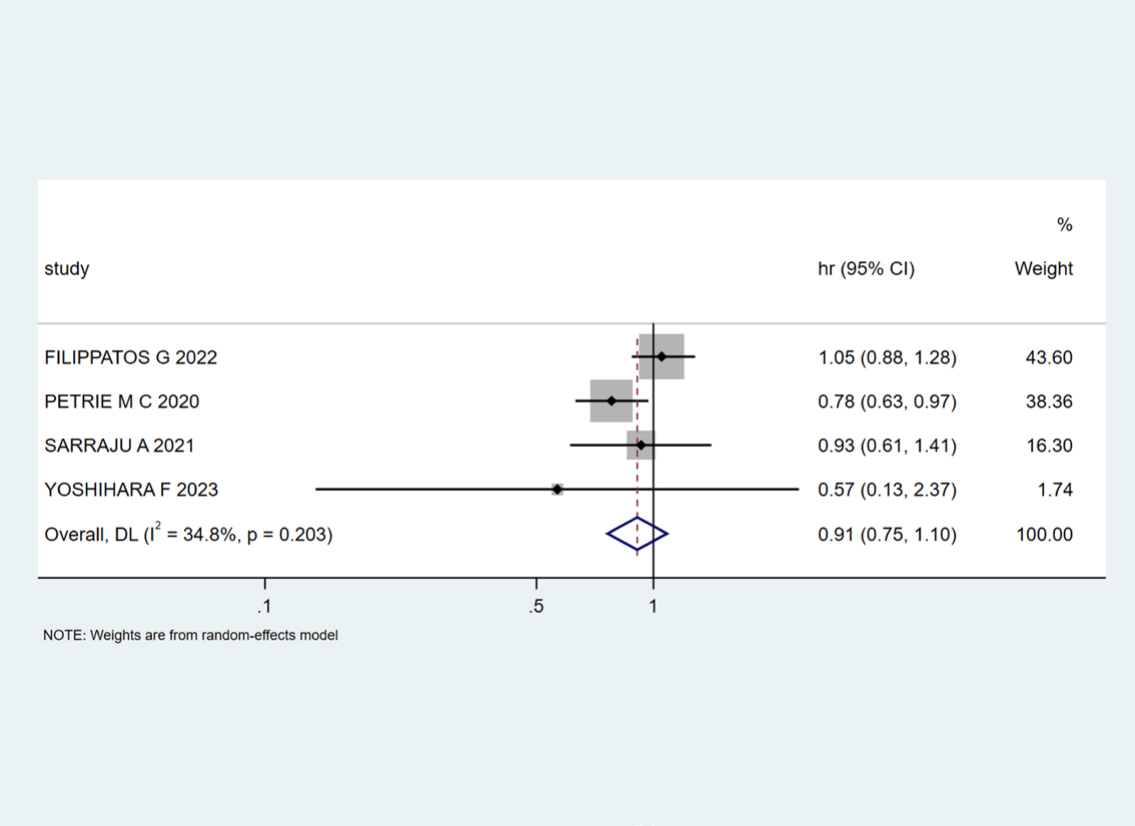
Impact of SGLT-2 inhibitors on the risk of all-cause mortality in patients. SGLT-2 = sodium-glucose cotransporter 2.

Four studies^[22,24,26,31]^ examined the time to all-cause mortality in patients with HF and T2DM treated with SGLT-2 inhibitors. Mild heterogeneity was present (I² = 34.8%, *P* = .203), necessitating the use of a random-effects model. The combined HR and 95% CI were 0.91 (0.75, 1.10), indicating a 9% reduction in all-cause mortality events with SGLT-2 inhibitor treatment (Fig. 9).

#### 3.2.3. Meta-analysis results of safety indicators

**Figure 10.**
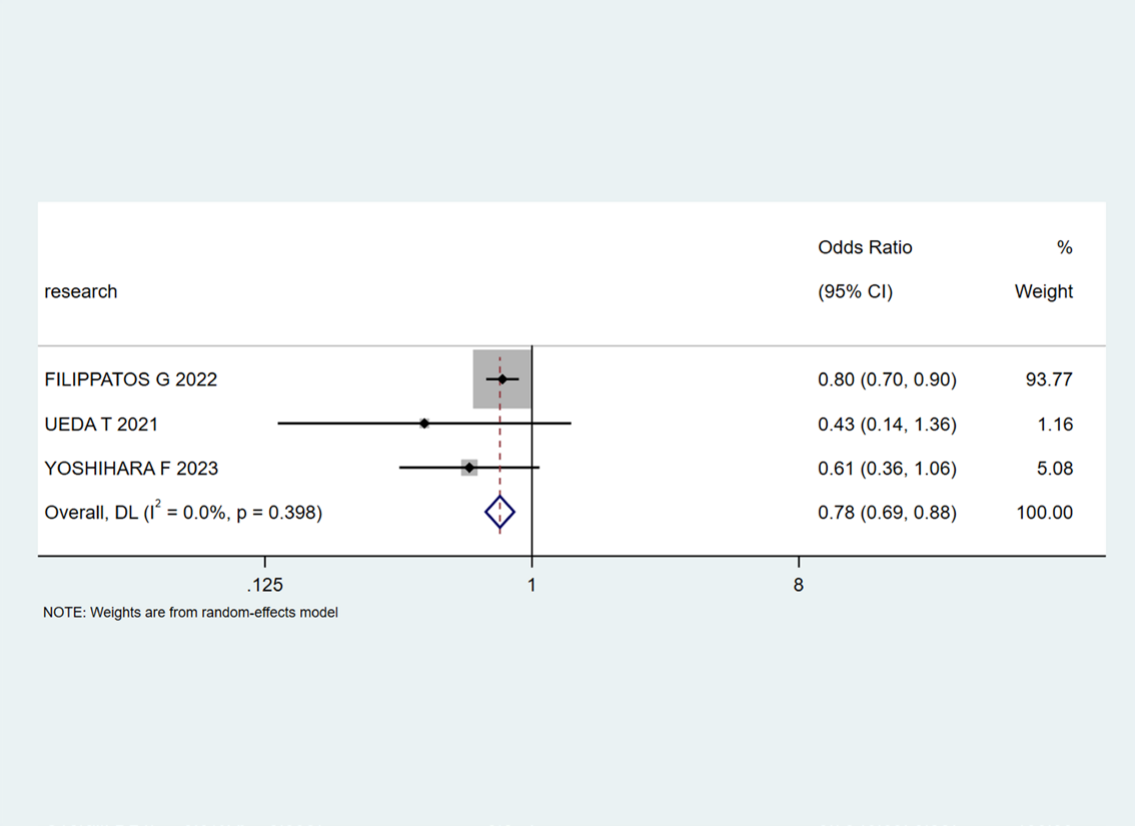
Impact of SGLT-2 inhibitors on the incidence of adverse reactions in patients. SGLT-2 = sodium-glucose cotransporter 2.

Three articles^[22,29,31]^ mentioned the incidence of adverse reactions. Heterogeneity analysis showed I² = 0%; therefore, the fixed-effects model was selected. The results of the meta-analysis showed that the combined effect size OR = 0.78, 95% CI (0.69, 0.88), Z = 6.55, *P* < .00001, indicating that the incidence of adverse reactions in the experimental group was significantly lower than that in the control group, with statistical significance, as shown in Figure 10.

## 4. Discussion

SGLT-2 inhibitors, or sodium-dependent glucose transporter 2 (SGLT-2) inhibitors, are a new type of hypoglycemic drug. Their successful development has provided a new approach for the treatment of diabetes and has become a research hotspot for hypoglycemic drugs.^[32,33]^ SGLT-2 is the primary transporter responsible for the reabsorption of glucose filtered by renal tubules. It is distributed in the S1 segment of the proximal renal tubules and completes the reabsorption of approximately 90% of the glucose in the glomerular filtrate.^[34]^ SGLT-2 inhibitors can selectively inhibit SGLT-2 receptors and reduce glucose reabsorption in the proximal renal tubules.^[35]^ By inhibiting SGLT-2 receptors, SGLT-2 inhibitors can lower the renal threshold for glucose and allow glucose that would otherwise be reabsorbed in the kidneys to be excreted in urine, thereby increasing urinary glucose excretion and reducing blood glucose levels.^[36,37]^ With the increase in urinary glucose excretion, the glucose concentration in the blood gradually decreases, achieving a hypoglycemic effect. This hypoglycemic method does not depend on insulin secretion or sensitivity; therefore, it is also effective in diabetic patients with insulin resistance.^[38,39]^ Currently, various SGLT-2 inhibitors have been launched globally, such as dapagliflozin, empagliflozin, and canagliflozin. Among them, dapagliflozin has been approved by the China Food and Drug Administration and is available in China.

Heart failure complicated by T2DM is a complex clinical condition that requires comprehensive consideration of multiple factors in its treatment. Among the included literature, 4 indicators frequently appeared: LVEDVI, BNP, KCCQ, and NT-proBNP. These 4 indicators comprehensively evaluate the therapeutic effects of SGLT-2 inhibitors in patients with HF complicated by T2DM from the aspects of cardiac function and quality of life. The results of this meta-analysis showed that the experimental group treated with SGLT-2 inhibitors demonstrated significantly better outcomes than the control group in terms of LVEDVI, BNP, KCCQ, and NT-proBNP, indicating that SGLT-2 inhibitors can be an effective treatment for patients with HF complicated by T2DM. LVEDVI is an indicator that measures the left ventricular volume at the end of diastole, reflecting cardiac diastolic function.^[40]^ SGLT-2 inhibitors promote urinary glucose excretion and reduce blood volume, thereby decreasing cardiac preload, contributing to improved ventricular diastolic function, and subsequently, reducing LVEDVI. Furthermore, SGLT-2 inhibitors may also enhance cardiac efficiency by improving energy metabolism in cardiomyocytes, further reducing the end-diastolic ventricular volume.^[41]^ BNP and NT-proBNP are peptide hormones secreted by the heart, are closely related to increased cardiac volume and pressure load, and serve as important biomarkers for HF diagnosis and prognosis assessment.^[42]^ The significant efficacy of SGLT-2 inhibitors in HF treatment may be partially attributed to their regulation of BNP and NT-proBNP levels. Although the specific mechanisms are not fully understood, SGLT-2 inhibitors may reduce the secretion of BNP and NT-proBNP by improving cardiac function and decreasing cardiac load. Additionally, SGLT-2 inhibitors may further lower BNP and NT-proBNP levels by inhibiting myocardial fibrosis and improving cardiac remodeling.^[43]^ The KCCQ is a crucial tool for assessing the quality of life in HF patients, encompassing dimensions such as symptom assessment and functional status. The significant improvement in KCCQ scores with SGLT-2 inhibitors suggests that these drugs not only enhance cardiac function but also improve patients’ quality of life.^[44]^ This may be related to the role of SGLT-2 inhibitors in reducing HF symptoms, improving exercise tolerance, and decreasing rehospitalization rates.^[45]^ Studies have also indicated that SGLT-2 inhibitors may indirectly enhance patients’ quality of life by improving their psychological state, such as by reducing anxiety and depression.^[46]^

Heart failure and T2DM are significant contributors to all-cause mortality. Heart failure affects at least 26 million people globally, with an increasing prevalence every year.^[47]^ A study of HF patients with T2DM revealed that hypoglycemic events are a risk factor for cardiovascular adverse events and increased all-cause mortality.^[48]^ In the CHARM trial, even after adjusting for multiple covariates, diabetes remained an independent risk factor for all-cause mortality in HF patients.^[49]^ Therefore, patients with HF complicated with T2DM face a higher risk of cardiovascular death and all-cause mortality, and understanding the impact of SGLT-2 inhibitors on patient prognosis is crucial for improving their quality of life. The combined results of this meta-analysis indicated that the experimental group treated with SGLT-2 inhibitors experienced a 23% reduced risk of cardiovascular death or hospitalization for HF, a 19% reduced risk of cardiovascular death, and a 9% reduced risk of all-cause mortality. Multiple large randomized controlled trials, including EMPA-REG OUTCOME,^[50]^ NVAS,^[51]^ and CLARE-TIMI 58,^[52]^ have confirmed the significant effects of SGLT-2 inhibitors in reducing the risks of cardiovascular death, HF hospitalization, and all-cause mortality. Heart failure-specific trials, such as DAPA-HF^[53]^ and EMPEROR-Reduced,^[54]^ have also demonstrated that SGLT-2 inhibitors significantly reduce the risks of cardiovascular death and hospitalization in HF patients. Through mechanisms including glucose-lowering, cardiac function improvement, and anti-inflammatory and anti-fibrotic effects, SGLT-2 inhibitors reduce cardiovascular events and thereby improve patient prognosis. The results of this meta-analysis showed that the incidence of adverse reactions in the experimental group was significantly lower than that in the control group. SGLT-2 inhibitors for the treatment of HF complicated by T2DM significantly reduce the occurrence of adverse reactions, such as infections, fractures, diabetic ketoacidosis, hyperkalemia, and liver dysfunction, indicating a high level of safety.^[55,56]^ The long-term use of SGLT-2 inhibitors exhibits good tolerability and a low rate of adverse reactions, allowing patients to adhere to treatment with greater peace of mind. As clinical research continues to deepen and drug applications become more widespread, the position of SGLT-2 inhibitors in the management of HF complicated by T2DM will be further consolidated, providing more potent treatment options for clinicians and patients.

While the primary mechanism of action of SGLT-2 inhibitors is well understood, recent studies^[6,7,57]^ have expanded our understanding of their multifactorial effects. These studies suggest that beyond their glucose-lowering action, SGLT-2 inhibitors may reduce oxidative stress and improve endothelial function, which could explain their cardiovascular and renal benefits. However, further investigation is required to fully elucidate the molecular mechanisms underlying these effects, particularly in populations with cardiovascular comorbidities and chronic kidney disease.

This study provides comprehensive evidence supporting the efficacy and safety profile of SGLT-2 inhibitors in patients with HF complicated by T2DM. These findings highlight their potential to reduce cardiovascular events, improve HF symptoms, and enhance quality of life in this high-risk population. These results contribute to the growing body of knowledge on the multifaceted benefits of SGLT-2 inhibitors, underscoring their role not only as effective hypoglycemic agents but also as essential cardiovascular and renal protectors in patients with both HF and T2DM. In the context of future medical developments, the results of this meta-analysis can guide clinicians in optimizing treatment regimens for patients with the dual burden of HF and T2DM. The evidence here supports the broader implementation of SGLT-2 inhibitors in clinical practice, especially as adjunctive therapy for improving cardiac and metabolic outcomes. Moreover, this study strengthens the need for further research on the long-term effects of SGLT-2 inhibitors, particularly regarding their impact on HF progression and diabetic complications over extended treatment periods. However, several important gaps remain in the current evidence, limiting the full potential of SGLT-2 inhibitors in clinical practice. Future studies should address the the limitations as study design and heterogeneity: while meta-analysis synthesizes results from multiple randomized controlled trials, significant heterogeneity in study designs, population demographics, and intervention protocols poses a challenge to generalizability. More uniform study designs with consistent endpoints would help to consolidate this evidence.

Although SGLT-2 inhibitors have shown strong short-term efficacy, data on their long-term effects, especially in the context of older populations with multiple comorbidities, are still lacking. Extended follow-up periods are necessary to evaluate the sustained benefits and potential risks, such as the impact on kidney function or cardiovascular outcomes over time. Currently, there is insufficient evidence to compare the efficacy of different SGLT-2 inhibitors in patients with HF and diabetes. This leaves a gap in the understanding of whether 1 agent might be more effective or safer than others in this specific population. While this study emphasizes the efficacy of SGLT-2 inhibitors as monotherapy, there is a pressing need to investigate the optimal combination of SGLT-2 inhibitors with other HF medications (such as ACE inhibitors, β-blockers, and aldosterone antagonists). This could further improve outcomes and enhance individualized treatment of patients with multiple health challenges. The majority of studies included in this meta-analysis focused on older, predominantly male, populations. Future research should aim to include a broader demographic to better understand the potential benefits and risks of SGLT-2 inhibitors in different age groups, genders, and ethnic backgrounds.

## 5. Conclusion

This study, through a systematic meta-analysis, synthesized data from multiple high-quality research studies, revealing the significant efficacy and good safety profile of SGLT-2 inhibitors in patients with HF complicated by T2DM. However, this study has several limitations. Firstly, the relatively small number of studies included, coupled with significant variations in study follow-up durations, may have impacted the interpretation of the results. Secondly, by focusing primarily on English-language literature, this study potentially overlooked important research published in other languages, limiting the comprehensiveness of the findings. Thirdly, further investigation is needed to explore the specific efficacy differences among different SGLT-2 inhibitors and potential drug–drug interactions. Future research should strive to conduct larger-scale, multicenter, randomized controlled trials to more accurately assess the efficacy and safety of SGLT-2 inhibitors in patients with various types of HF and T2DM. Additionally, exploring the combined use of SGLT-2 inhibitors with other HF treatments (such as ACEI/ARBs, β-blockers, and aldosterone receptor antagonists) is essential for optimizing treatment regimens. The findings of this meta-analysis strongly support the use of SGLT-2 inhibitors in patients with HF and T2DM, demonstrating significant reductions in mortality, hospitalization for HF, and improvement in quality of life. However, further research is needed to address these limitations, particularly in terms of long-term safety, comparative efficacy, and combination therapy approaches. Future investigations should aim to fill these gaps and refine treatment protocols, ultimately contributing to the evolving landscape of HF and diabetes management. With more comprehensive data, SGLT-2 inhibitors have the potential to become the cornerstone of therapeutic strategies in this patient population.

## Author contributions

**Conceptualization:** Xinliang Yao, Xueli Lu.

**Data curation:** Xinliang Yao, Han Zhang, Xueli Lu.

**Formal analysis:** Xinliang Yao, Han Zhang, Xueli Lu.

**Investigation:** Xinliang Yao, Xueli Lu.

**Methodology:** Xinliang Yao, Xueli Lu.

**Resources:** Xueli Lu.

**Software:** Xinliang Yao, Han Zhang, Xueli Lu.

**Supervision:** Xinliang Yao.

**Validation:** Xinliang Yao, Han Zhang.

**Visualization:** Xinliang Yao, Han Zhang.

**Writing – original draft:** Xinliang Yao, Han Zhang, Xueli Lu.

**Writing – review & editing:** Xinliang Yao, Xueli Lu.
